# Long-Term Examination of Water Chemistry Changes Following Treatment of Cyanobacterial Bloom with Coagulants and Minerals

**DOI:** 10.3390/ijerph192013577

**Published:** 2022-10-20

**Authors:** Bokjin Lee, Heejun Kang, Hye-cheol Oh, Jaehwan Ahn, Saerom Park, Sang-Leen Yun, Seogku Kim

**Affiliations:** 1 Civil and Environmental Engineering, University of Science and Technology (UST), Daejeon 34113, Korea; 2 Department of Environmental Research, Korea Institute of Civil Engineering and Building Technology (KICT), Goyang 10223, Korea

**Keywords:** cyanobacteria removal, coagulation materials, nutrient release, eutrophication, sedimentation

## Abstract

The abundant growth in cyanobacterial blooms poses severe ecological threats with a high risk to aquatic organisms and global public health. Control of cyanobacterial blooms involves spraying cyanobacteria removal materials, including coagulants. However, little is known about the fate of the coagulated-cyanobacteria-laden water. Here, we examined long-term changes in water quality following treatment with various coagulants and minerals for cyanobacterial removal when the coagulated cyanobacterial cells were not removed from the water. An experiment in a controlled water system tested the effects of six different compounds, one conventional coagulant, two natural inorganic coagulants, and three minerals. All tested coagulants and minerals exhibited >75% of cyanobacterial removal efficiency. However, compared to the control, higher concentrations of nitrogen were observed from some samples treated during the experimental period. After 20 months, the final total phosphorus concentration of the raw water increased 20-fold compared to the initial concentration to 11.82 mg/L, indicating significant nutrient release over time. Moreover, we observed that the decomposition of sedimented cyanobacterial cells caused the release of intracellular contents into the supernatant, increasing phosphorous concentration over time. Therefore, cyanobacterial cells should be removed from water after treatment to prevent eutrophication and maintain water quality.

## 1. Introduction

Cyanobacterial blooms, which are the overgrowth of cyanobacteria, also known as blue-green algae, occurs worldwide and poses a serious threat to the management of water bodies. The phenomenon is stimulated by anthropogenic factors such as climate change, fossil fuel use, and eutrophication caused by excessive fertilizer use and livestock wastewater runoff [1,2]. Since some of the cyanobacteria species produce toxins, increased amounts of potentially toxic cyanobacterial biomass not only affect the food web but limit aquatic activities [3,4]. The occurrence of cyanobacterial blooms also leads to problems in water treatment plants such as filter clogging, a need for increased coagulant dosages, and additional costs for advanced treatment processes for removing cyanobacterial metabolites [5,6].

A common method used to reduce cyanobacteria in fresh waterbodies is the direct dispersal of chemicals such as algaecides. Hydrogen peroxide (H_2_O_2_) and copper sulfate (CuSO_4_) are well-documented algaecides that suppress cyanobacterial blooms by inhibiting cell viability [7,8,9,10]. However, applying algaecides can potentially harm aquatic organisms and cause the release of cyanobacterial metabolites, such as cyanotoxins and taste-and-odor compounds [11,12,13]. In South Korea, the Enforcement Decree of the Water Environment Conservation Act of 2022 recommends “spraying substances for removing algae and taking any other measures to remove cyanobacteria”. Here, the most common choice of material for cyanobacteria removal is loess [14]. However, the mitigation of cyanobacterial blooms using minerals is temporary and thus requires repeated application, which can negatively impact water quality, such as microbes utilizing oxygen to decompose sedimented cyanobacterial cells, creating hypoxic dead zones where neither fish nor plants can survive [15]. Furthermore, the decomposition of sedimented cyanobacterial cells causes the release of intracellular content, which acts as the internal load that accelerates the cycle of eutrophication, cyanobacterial bloom occurrence, and water quality degradation [16]. Furthermore, cyanobacterial bloom treatment with natural or modified minerals could cause secondary pollution from organic matter or heavy metals [17].

Some studies report that the application of coagulants does not damage cyanobacterial cells; however, there are studies that highlight that coagulants disrupt cell integrity, resulting in the release of intracellular content [18,19,20]. The exposure time to the chemicals influences cyanobacterial cell integrity [19,21]. The reaction time in some studies was only several hours, which does not reflect actual conditions in water bodies; once coagulants or minerals are introduced, coagulated cyanobacterial cells remain in the water unless removed via additional measures, such as dredging. Additionally, little is known about how the treatment of cyanobacterial bloom affects water chemistry over a long period of time. Being able to monitor and predict potential changes in water quality over time is essential for effective water quality management. Therefore, when coagulants and minerals are used for cyanobacteria removal, the changes in water chemistry from coagulated-cyanobacteria-laden water must be considered for better insight into water quality management.

The objective of this study was to examine long-term changes in water quality following treatment with various coagulants and minerals for cyanobacterial removal, considering that the coagulated cyanobacterial cells are not removed from the water. A conventional coagulant, two natural inorganic coagulants, and three minerals were assessed for their effectiveness at removing cyanobacterial cells using samples from a cyanobacterial bloom in a eutrophic reservoir in South Korea. The nutrient concentrations of samples treated with either the coagulant or the mineral were analyzed monthly for two years. The findings of this study provide insights into the effect of the direct addition of coagulants and minerals into bodies of water on water quality.

## 2. Materials and Methods

### 2.1. Raw Water Sampling

The raw water for this study was obtained from the Shingal reservoir in Yongin City, Republic of Korea. The sampling point is an agricultural reservoir with an area of 2.31 km^2^, a total capacity of 11,659,000 m^3^, and a drainage basin area of 52.3 km^2^. The Shingal reservoir drainage basin has a population of approximately 250,000. There are 198 wastewater discharge facilities near the reservoir that discharge approximately 7300 m^3^ of wastewater per day. In addition, 35,000 tons of sewage effluent from nearby sewage treatment plants flows into the reservoir per day, contributing to eutrophication. The Shingal reservoir experiences intense cyanobacterial blooms every year. The raw water was sampled when there was excessive cyanobacterial growth on the surface of the reservoir, with a chlorophyll-a concentration of 48 mg/m^3^ (Table 1). Over 100 L of surface water was collected with a container that was cleaned with distilled water and stored in 20 L carboys. The carboys were transported to the laboratory for further experiments. At the laboratory, the raw water from each carboy was filtered using a 20-mesh sieve (850 μm) to remove debris and was then poured into a large basket and mixed thoroughly.

### 2.2. Coagulants and Minerals

We selected total of six different coagulants and minerals. Polyaluminum chloride (PACl), commonly used in drinking water treatment facilities, was obtained from Daemyung Chemical Co., Ltd. (Hwaseong-si, Korea). Two commercial coagulants were used in this study: mineralized coagulant (MC) was manufactured with natural minerals, sea water, and pine needle extract by researchers at the Korea Institute of Civil Engineering and Building Technology (Korea patent No. 10-2017-0100298), and coagulant A was purchased from GCM Korea (Cheonan-si, Korea). Three natural inorganic minerals, loess, sericite, and illite were purchased from a local distributor. All the characteristics and major compositions of each coagulant and mineral are presented in Table 2.

### 2.3. Jar Test

Optimum dosages of each coagulant and mineral were determined using a jar test apparatus with six paddle stirrers. In six beakers containing 500 mL of raw water, each coagulant and mineral was added with different dosages. The range of dosages varied in each coagulant and mineral; For liquid types, it was from 0.5 mL/L to 3 mL/L with 0.5 mL difference, and it was from 0.5 g/L to 5 g/L with 0.5 g of difference. The contents were rapidly mixed for 2 min at 150 rpm, followed by slow mixing for 20 min at 35 rpm. Chlorophyll-a concentration is used as an algal biomass indicator; in addition, it reflects the trophic state of the water body [22]. The concentration of chlorophyll-a in the supernatant was measured following 1 h of sedimentation, allowing the optimum dosage with the lowest chlorophyll-a value to be determined, and these steps were repeated six times for all coagulants and minerals. The optimum dosage of MC and PACl was 1 mL/L. The best cyanobacteria removal efficiencies for coagulant A and loess, sericite, and illite were observed at 2 g/L, 2 g/L, 4 g/L, and 1 g/L, respectively. For minerals, there was no significant difference in the amount of chlorophyll-a removed, even with increased dosage; therefore, the smallest dosage was selected. To mimic the conditions of direct dispersal of coagulants and minerals into natural bodies of water, the pH was not controlled.

### 2.4. Experimental Methods

Cylindrical test columns with a diameter of 160 mm and a height of 600 mm were prepared (Figure S3). All columns were washed with distilled water and then with raw water to reduce errors caused by impurities. Seven columns were filled with raw water, and each coagulant or mineral was added to its own column, with the seventh column serving as a control. To ensure sufficient reaction between the cyanobacteria and coagulant or mineral, all the columns were mixed for 10 min using a portable stirrer. The columns were then left undisturbed at room temperature (15 °C–25 °C). To prevent contamination and evaporation, the columns were closed with lids but were not completely sealed. The supernatant from each column was sampled every month for 20 months. The pH, turbidity, total nitrogen (TN), total phosphorus (TP), ammoniacal nitrogen (NH_3_-N), nitrate nitrogen (NO_3_-N), and phosphate (PO_4_-P) levels of each sample were analyzed. At the end of the experiment, the sedimented cyanobacterial cells were carefully removed for cell integrity analysis.

### 2.5. Analytical Methods

TN and TP were measured using a UV/Visible spectrophotometer (DR 5000, HACH Corp., Loveland, CO, USA), according to Standard Methods for the Examination of Water and Wastewater, 22nd edition. pH and turbidity were measured using a pH meter (Orion Star A111, Thermo Scientific, Waltham, MA, USA) and a turbidimeter (2100N, HACH Corp. Loveland, CO, USA), respectively. TN and TP were analyzed according to UV/Visible spectrometry methods outlined by the Standard Method for the Examination of Water Pollution established by the Ministry of Environment (ES 04363. 1a and ES 04362. 1c). The dissolved nutrients (NO_3_-N, NH_3_-N, and PO_4_-P) were analyzed according to Standard Methods for the Examination of Water and Wastewater, 22nd edition, using an Ion Chromatography system (ICS-3000, Thermo Scientific). Cell integrity was assessed using Sytox^®^ Green (Thermo Fisher Scientific; 2 nM) with a laser scanning confocal microscope (ZEISS, LSM 700, Jena, Germany), as described in previous studies [23,24].

## 3. Results and Discussion

### 3.1. Cyanobacterial Removal Efficiencies of the Coagulants and Minerals

The coagulation and flocculation efficiencies of the coagulants and minerals were assessed based on the chlorophyll-a concentrations before and after the treatment. Chlorophyll-*a* removal efficiencies of all coagulants and minerals except sericite were higher than 90%. PACl was the most efficient at 98.5%, followed by illite (97.7%), mineralized coagulant (MC) (96.0%), loess (95.6%), and coagulant A (92.7%). At 1.2 mL/L, PACl has been shown to remove green algae (*Chlorella* sp. and *Monoraphidium* sp.) at 99.7% efficiency, where the chlorophyll-a concentration of the tested water was 5325 mg/m^3^ [25], which is consistent with the results of our study. According to Choi, raw sericite, a hybrid material for algae removal, was shown to have a chlorophyll-a removal efficiency of 24% when the average chlorophyll-*a* concentration was 62.3 mg/m^3^ [14]. However, in our study, sericite exhibited 74.8% chlorophyll-*a* removal efficiency. The difference between the two studies could be attributed to variations in raw water characteristics and experimental procedures.

Liquid coagulants were more efficient in removing chlorophyll-*a* than powder-form minerals. The main floc formation mechanism of liquid coagulants is charge neutralization; the cations in the coagulants neutralize the negatively charged cyanobacterial cells [26]. In contrast, minerals exhibit low solubility or are insoluble; therefore, the key cyanobacterial cell removal mechanisms are physical aggregation and precipitation [26,27]. Additionally, most minerals have negative surface charges that hinder adsorption to the negatively charged cyanobacterial cells [28,29]. Hence, charge neutralization occurs in liquid coagulants and shows greater coagulation efficiency than physical adsorption. However, unlike other inorganic particulate matter, some species of cyanobacteria have a gelatinous outer coating composed of extracellular organic matter that enhances cell adhesion and promotes the adsorption of solid minerals. This could explain the high chlorophyll-*a* removal efficiencies of loess and illite [30,31] (see also Issa et al. 2001). Sericite expands in polar liquids and is negatively charged. This explains its lower efficiency than the two other minerals [32].

### 3.2. Changes in Water Chemistry Following Treatment with Coagulants and Minerals

#### 3.2.1. Turbidity

The turbidity of the control column was initially high at 3924 NTU but decreased over time, with 15 months elapsing before full stabilization, which was the reason for selecting 20 months as the analysis period to fully investigate the water chemistry changes (Figure S1). MC was the only coagulant that caused the flotation of the cyanobacteria. The cyanobacteria were present at the surface of the raw water for two months, after which they settled to the bottom of the column. The floating cyanobacteria flocs settled to the lower layer, causing desorption and thereby increasing the turbidity. No further change in turbidity was observed in subsequent analyses; therefore, flocs do not appear to have desorbed after sedimentation.

The differences in chlorophyll-*a* removal efficiencies and turbidity among the minerals tested could be attributed to differences in the specific gravity and settling time of each mineral. The specific gravities of loess, illite, and sericite were 2.667, 2.71−2.8, and 2.35, respectively [33,34,35,36]. The lower the specific gravity, the longer it takes for the cyanobacterial cells to settle. To avoid disrupting coagulated cyanobacterial cells, only the supernatants were sampled; however, minerals with a lower specific gravity would have required more time for complete sedimentation. This is reflected in the turbidity values recorded after one month, since the column with sericite, which has the lowest specific gravity of the three minerals, had the highest turbidity.

This study used a controlled water system that was not affected by environmental factors. Natural bodies of water have more environmental variables, such as wind-generated waves, rainfall, drainage basins, and aquatic biological activities. Therefore, it would be nearly impossible to perfectly stabilize the cyanobacteria and coagulant flocs in real world conditions. Wind-induced resuspension of flocs or sediments, particulate organic matter, and nutrients alters water chemistry in natural water bodies. When coagulants and minerals are applied to shallow bodies of water, the flocs can easily become resuspended by wind. Both the unexposed and exposed parts of the flocs are revealed and interface with the water, which eventually causes the dispersion of cyanotoxins, organic matter, and nutrients [37]. Turbidity can be used as a measure of floc and sediment resuspension. However, an increase in turbidity does not always indicate an increase in nutrients because an equilibrium is established between the water and flocs or sediments in the presence of nutrient-absorbing entities [38]. The stable turbidity values in this study suggest that there was no significant physical interference from the external environment. Therefore, changes in water chemistry were driven by the coagulants and minerals.

#### 3.2.2. Nutrients

##### Nitrogen

**Figure 1:**
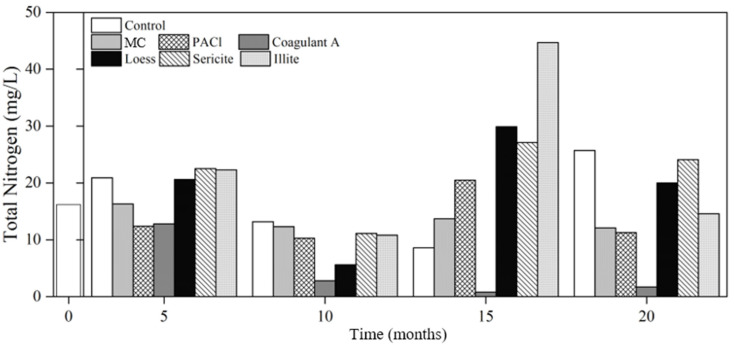
Changes in total nitrogen concentration in the control and treatments with various coagulants and minerals. MC: mineralized coagulant; PACl: polyaluminum chloride; A: commercial coagulant.

Nitrogen is necessary for protein synthesis in cyanobacterial cells, modulating their growth and cyanotoxin production [39,40]. To study the effect of coagulants and minerals on nutrient concentrations in cyanobacterial cells, different nitrogen and phosphorus forms were analyzed. The initial TN and NO_3_-N concentrations were 18.2 mg/L and 0.09 mg/L, respectively. The NH_3_-N concentrations were not detected. The TN concentrations of the control increased slightly in the 5th analysis (after five months of the coagulants and minerals addition), decreased until the 15th analysis to 8.60 mg/L, and then increased in the 20th analysis to 25.7 mg/L, which was 58.6% higher than the initial concentration (Figure 1). The TN concentrations in the loess, sericite, and illite columns increased over time, becoming higher with each analysis. Nitrogen is released from loess upon addition to water [41]. Since sericite and illite have a similar composition to loess, such increase in TN concentrations occurred. Therefore, the use of minerals and soils in cyanobacterial bloom control may likewise introduce nitrogen loads to the water bodies.

Coagulant A was the most effective in reducing TN concentrations; the TN concentrations in the 10th, 15th, and 20th analyses were 2.8 mg/L, 0.8 mg/L, and 1.7 mg/L, respectively. Coagulant A is a combination of different minerals. In water, as the oxygen binds to each mineral dissociate, cations such as Al^3+^ and Ca^2+^ chemically bind to these minerals. Therefore, TN concentration in the Coagulant A column was stably low for almost two years.

In the 15th analysis, the TN concentration in the PACl (20.5 mg/L) column was 2.86 times higher than that in the control (8.6 mg/L). Despite its good chlorophyll-*a* removal efficiency, PACl damages cyanobacterial cells. This results in the release of intracellular organic matter, which could be responsible for the increase in TN concentration [19].

**Figure 2:**
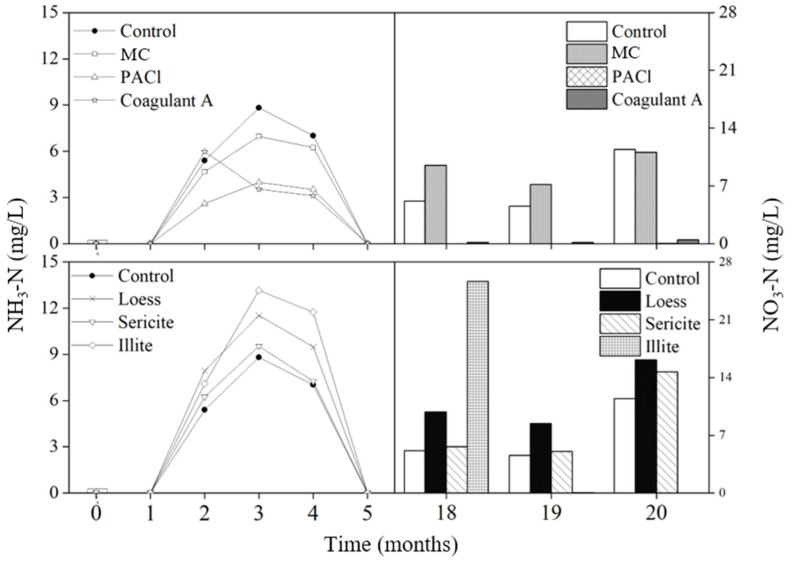
Changes in NH3-N and NO3-N concentrations in the control and treatment columns with coagulants and minerals. MC: mineralized coagulant; PACl: polyaluminum chloride; A: commercial coagulant.

TN concentration represents the sum of all soluble/particulate and organic/inorganic forms of nitrogen. The NH_3_-N concentration spiked in all columns two months after the addition of coagulants and minerals (Figure 2). MC and PACl treatments showed lower NH_3_-N concentrations than the control, while all mineral treatments exhibited higher NH_3_-N concentrations than the control. High ammonia concentration in the first few analyses implies the release of ammonia from dead cyanobacterial cells as they decompose [42]. Oxygen consumption during cellular decomposition promotes the denitrification process, which occurs under anaerobic conditions. Therefore, it is possible that coagulants could impede the decomposition of cells, however further microbial analysis should be accompanied to understand the mechanism After the 4th analysis, only a low concentration of NH_3_-N was detected in some columns.

Under aerobic conditions in water, nitrogen changes from ammonia to nitrite and eventually to nitrate. Considering this cycle, increased nitrate concentrations detected in the 18th analysis indicate that it took 18 months for nitrification to occur under these particular experimental conditions (Figure 2). The columns were covered with lids to avoid contamination; however, they were not completely sealed. Therefore, the supernatant of the samples was exposed to some amount of air, enabling some aerobic microbial growth. Four months after the release of NH_3_-N due to lysis, an equilibrium was achieved between nitrification and denitrification. Both NH_3_-N and NO_3_-N were not detected until the 18th analyses [43]. Then, conditions in the control, MC, loess, and sericite columns became favorable for nitrifying bacteria; this resulted in increasing NO_3_-N concentrations at the end of the experiment. Significantly low NO_3_-N conditions were observed in the PACl and Coagulant A columns. PACl severely inhibits hydrolysis, acidogenesis, and methanogenesis, which are involved in the anaerobic fermentation of sludge [44]. Although our study focused on cyanobacterial cells and the columns were not fully anaerobic, PACl may have inhibited the activation of nitrifying bacteria, resulting in low nitrate concentrations. The non-detection of NH_3_-N indicated the inhibition of floc solubilization by PACl. Further analysis of the microbial community would help to better understand the bacterial mechanism in water quality changes.

##### Phosphorus

**Figure 3:**
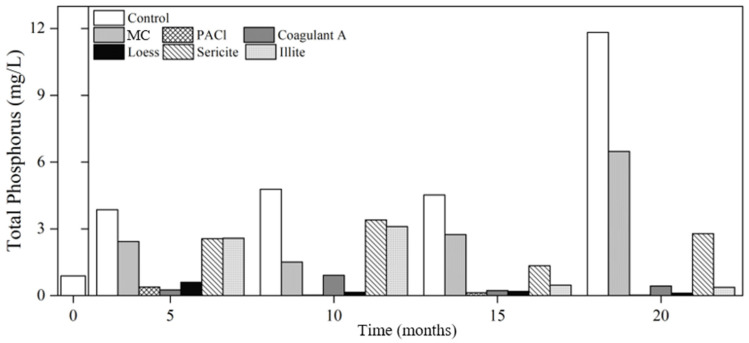
Changes in total phosphorus concentrations in the control and treatments with coagulants and minerals. MC: mineralized coagulant; PACl: polyaluminum chloride; A: commercial coagulant.

Phosphorus is one of the most limiting nutrients for cyanobacterial growth; controlling phosphorus is important for managing eutrophication [45,46]. The initial TP concentration of the raw water was 0.88 mg/L (Figure 3). After five months of not removing the cyanobacteria from the column, TP increased to 3.93 mg/L, which was 4.5 times higher than the initial concentration. The TP concentration of the control increased until the last analysis at 20 months when it was 11.82 mg/L, which is 13 times higher than the initial concentration. This increase over time could be related to sedimentation. At the beginning of the experiment, raw water was distributed uniformly in each column with continuous stirring; the volume of cyanobacteria was assumed to be comparable between columns. The density of the coagulated and flocculated cyanobacteria in the treatment columns was increased because of the specific gravity of the added coagulants and minerals. The cyanobacterial flocs compressed and settled over time. Therefore, the relatively loosely compressed cyanobacterial cells in the control had more specific surface area than those in columns treated with coagulants and minerals and thus had more interface with the water and a higher chance of releasing nutrients into the supernatant. In contrast, relatively low TP concentrations in the coagulant and mineral samples could be the result of adsorption and formation of cation–phosphate complexes. Additionally, bacteria play an important role in the phosphorus cycle, so their activity might have been different in each sample which resulted in different phosphorus concentrations.

The coagulants and minerals that exhibited the least release of phosphorus were PACl, coagulant A, and loess. The phosphorus removal efficiency of PACl is well established; the amorphous Al(OH)_3_, with high affinity in water strongly binds with phosphate [47]. The binding of PACl with nutrients and the inhibition of floc solubilization likely resulted in low phosphorus and nitrogen concentrations. The phosphorus removal efficiency of loess at the 20th analysis was 87.5%, which is consistent with previous reports of high phosphorus adsorption capacity of loess [48,49]. A study of phosphorus compounds in sediments revealed that, apart from dissolved forms, PO4- and organic P, particulate forms such as Fe (III) hydroxides, ferric oxyhydroxide (Fe(OOH)), strengite (FePO_4_), vivianite (Fe_3_(PO_4_)_2_8H_2_O), aluminum hydroxide (Al(OH)_3_), variscite (AlPO_4_), hydroxyapatite (Ca_10_(PO_4_)_6_OH_2_), monetite (CaHPO_4_), and calcite (CaCO_3_) were present [38]. The cations in Coagulant A and minerals (i.e., loess, sericite, and illite) could bind with phosphorus to form these compounds, resulting in low TP concentration [50]. Although there was a substantial P increase in the MC-treated sample at the 20th analysis, MC was developed for use with a microbubble-generation system to remove cyanobacteria through flotation and separation, enabling cyanobacteria to be easily skimmed from the top of the water. Therefore, it is expected that nutrient release would not occur when cyanobacterial cells are removed from the water via this coagulation method which should be studied in the future for in-depth understanding of possible water quality change.

A body of water with a TP concentration over 0.02 mg/L is considered eutrophic [51,52]. The highest concentration was observed in the control at 20th analysis and was 11.82 mg/L, which was 591 times higher than the initial concentration. This experiment was conducted in a closed system without external environmental influence; however, the high TP concentration proved that nutrient release could occur at alarming levels in natural bodies of water.

**Figure 4:**
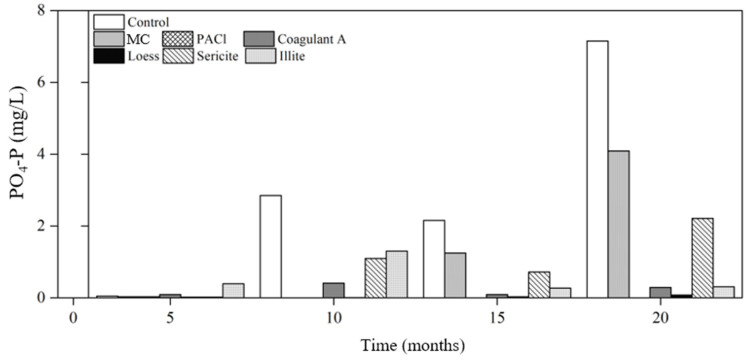
Changes in phosphate concentrations in the control and the treatments with various coagulants and minerals. MC: mineralized coagulant, PACl: polyaluminum chloride, and A: commercial coagulant.

Non-detection of phosphate in raw water indicates uptake of phosphate by cyanobacterial cells; phosphorus was the limiting factor in cyanobacterial growth in the Shingal reservoir. Considering TP concentrations, notably low concentrations of PO_4_-P were detected in the 5th analysis, implying that most of the phosphorus existed in particulate form (Figure 4). PO_4_-P was detected after the 5th analysis; its concentration was the highest in the control at 2.85 mg/L, 2.16 mg/L, and 7.15 mg/L in the 10th, 15th, and 20th analyses, respectively.

The change in the phosphorus form from particulate to dissolved in open water bodies is a serious problem in terms of eutrophication and cyanobacterial blooms because phosphates are utilized by cyanobacteria. The increasing proportion of PO_4_-P in the TP measurements demonstrates the importance of nutrient control (Figure S2). A study conducted in a hypereutrophic lake revealed that most of the phosphorus released from cyanobacteria decomposition exists in the organic form rather than as inorganic dissolved phosphorus [53]. Although organic phosphorus concentration was not analyzed, the results of our study are consistent with this finding because the level of phosphate was very low (0.05 mg/L), even with a high TP concentration of 3.86 mg/L. The proportion of phosphate continued to increase in all columns over time such that over 60% of phosphorus was quantified as phosphate, except in the PACl column, in which phosphate was not detected after the 5th analysis. Compared to the control in which over 50% of phosphorus existed in phosphate form, the samples treated with coagulants and minerals exhibited a relatively low proportion of phosphate until the 15th analysis. However, in the 20th analysis, PO_4_-P/TP in all columns except the PACl column exceeded the control, with up to 83.8% in the illite column. This again demonstrates the importance of long-term monitoring of water chemistry.

In natural water bodies, the form of phosphorus can vary by season. For example, most phosphorus exists as orthophosphate during winter, which coincides with the death of cyanobacteria [54]. Considering that cyanobacterial blooms occur during summer, sedimented cyanobacterial cells would increase phosphate concentration in the water body in the following winter, and the cells would contribute to internal phosphorus loading. Hence, the increased proportion of phosphate arising from cyanobacterial cells signifies that the longer treated cyanobacterial cells remain in water, the higher the chance of cyanobacterial phosphorus assimilation. However, given the much lower phosphate proportions of the treated samples in the 5th analysis when compared to the control, the coagulants and minerals used to aggregate cyanobacterial cells and then remove them immediately is promising for managing eutrophication because of the decreased chance of nutrient release.

### 3.3. Effects of Coagulants and Minerals on Cell Integrity

**Figure 5:**
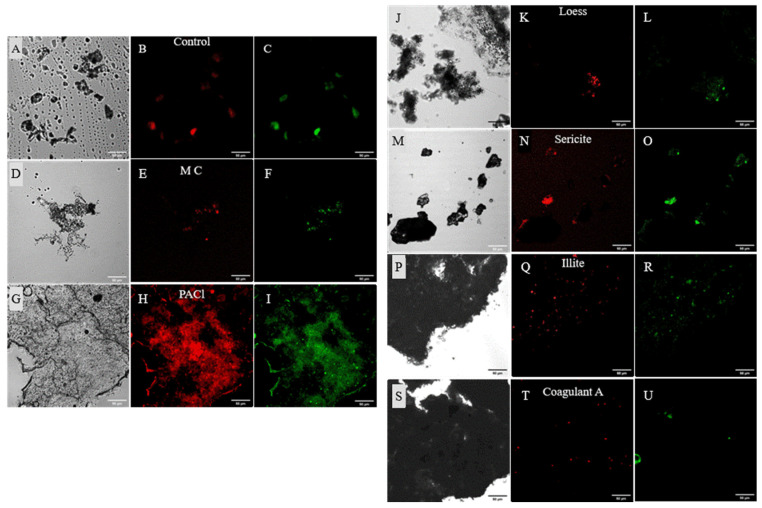
Images of cyanobacterial cells in the control (**A**–**C**), MC (**D**–**F**), PACl (**G**–**I**), Loess (**J**–**L**), Sericite (**M**–**O**), Illite (**P**–**R**), and Coagulant A (**S**–**U**). Each image of (**A**,**D**,**G**,**J**,**M**,**P**,**S**) shows bright-field, (**B**,**E**,**H**,**K**,**N**,**Q**,**T**) red autofluorescence of cells, and (**C**,**F**,**I**,**L**,**O**,**R**,**U**) SYTOX^®^ Green staining. Cells with a green fluorescence color show that there is a cell damage indicating that the dye bound to nucleic acid. Scale bar = 50 µm. MC: mineralized coagulant; PACl: polyaluminum chloride; Coagulant A: commercial coagulant.

Coagulants and algaecides damage cyanobacteria cells, resulting in the release of nutrients and intracellular cyanobacterial metabolites and thus promoting further water pollution and eutrophication [20,37]. Nutrient release occurred even 20 months after coagulation and flocculation. Increased nutrient release from the coagulated and flocculated cyanobacterial cells indicates floc resuspension, potentially occurring as a result of compromised cell integrity, results in the presence of the particulate form of nutrients in the supernatant. Compromised cell integrity is caused by cell decomposition and cell damage from chemical exposure or physical deterioration in response to the coagulants and minerals. To confirm cell damage, cells were stained with SYTOX green; this dye selectively stains nucleic acids in the cell. Damaged cells are distinguished based on the fluorescence as their damaged cell membranes would enable the dye to penetrate the cell structures and stain nucleic acids [23,24]. The bright-field images (Figure 5A) show a varying number of cyanobacterial cells and flocs of different shapes in each sample. Cyanobacterial cells, which were visualized using SYTOX green, were difficult to locate since they were trapped between mineral particles. The cell integrity analysis revealed that cyanobacterial cells from the control were also damaged, and the stained nucleic acids were observed. This suggests that cyanobacterial cells should be removed from the water in the event of a cyanobacterial bloom; otherwise, they could cause internal nutrient load and cyanotoxin pollution. This analysis was only performed 20 months after the beginning of the experiment; in future studies, it would be informative to perform this analysis throughout the experimental period in order to gain insight into the effects of coagulants and minerals on cell integrity over time. At 20 months, cyanobacterial cells were damaged because of natural decomposition, interactions with the coagulants and minerals, or both. Intracellular content includes both nutrients and harmful compounds such as cyanotoxins. The coagulants and minerals had a positive effect on removing phosphorus; future studies should focus on their influence on cyanotoxin concentration.

This study provides new insights into managing cyanobacterial blooms with coagulants and minerals. Since the experiment was conducted in a controlled system without any effect of environmental factors, further studies should investigate how abiotic and biotic factors influence water chemistry in a natural environment.

## 4. Conclusions

The long-term examination of water chemistry after the treatment of cyanobacterial cells with various coagulants and minerals led to the following conclusions:

1. The phosphorus concentration of the raw water without removing cyanobacteria (control) increased by 4.5 times after 5 months (3.93 mg/L) and 13 times after 20 months (11.82 mg/L) compared to the initial concentration (0.88 mg/L). This increases the risk of eutrophication when cyanobacterial cells remained in the water. During the 20-month study period, the longer the cyanobacterial cells remained in the water, the more the phosphorus concentration increased, thus intensifying the risk of eutrophication.
2. The addition of coagulants and minerals enabled nutrient removal via complex formation and adsorption; however, the effect on other intracellular matter remains to be investigated.
3. Cell integrity analysis revealed cellular damage in all samples after 20 months. This highlights the importance of removing cyanobacterial cells from water to prevent eutrophication and cyanotoxin pollution.
4. Since the nutrient concentrations of the treated columns were lower than the control in the early stages, we propose the removal of coagulated and flocculated cyanobacterial cells immediately after treatment in order to reduce eutrophication and manage water quality.

## Figures and Tables

**Table 1:** Physical and chemical characteristics of the raw water in this study.

Parameter (Unit)	Value
pH	8.9
Turbidity (NTU)	3924
Chlorophyll-a (mg/m^3^)	48
Total nitrogen (mg/L)	16.2
Ammoniacal nitrogen (mg/L)	N.D.
Nitrate nitrogen (mg/L)	0.09
Total phosphorus (mg/L)	0.8
Phosphate (mg/L)	N.D.

N.D.: not detected.

**Table 2:** Major compositions of coagulants and minerals used in the study.

Coagulant and Minerals	Phase	Major Components
MC	Liquid	Na, Ca, Mg, K, Al, Cl, and pine needle extract
PACl	Liquid	Al_2_(OH)_3_Cl_3_ and Al_2_O_3_
Coagulant A	Powder	CaO, Al_2_O_3_, MgO, K_2_O, Fe_2_O_3_, and SiO_2_
Loess	Powder	Al_2_O_3_ and Fe_2_O_3_
Sericite	Powder	SiO_2_, Al_2_O_3_, CaO, and MgO
Illite	Powder	SiO_2_, Al_2_O_3_, Fe_2_O_3_, and K_2_O

## Data Availability

Not applicable.

## Supplementary Materials

The following supporting information can be downloaded at: https://www.mdpi.com/article/10.3390/ijerph192013577/s1, Figure S1: Changes in the turbidity values of control and samples treated with coagulants and minerals. MC: mineralized coagulant; PACl: polyaluminum chloride; A: commercial coagulant; Figure S2: Changes in phosphate concentrations in the control and the treatments with various coagulants and minerals. MC: mineralized coagulant; PACl: polyaluminum chloride; A: commercial coagulant; Figure S3: The experimental columns used in the study. The photo was taken at the end of the experiment.
