# Supplementary material for: Long-Term Examination of Water Chemistry Changes Following Treatment of Cyanobacterial Bloom with Coagulants and Minerals
Source: Int J Environ Res Public Health. 2022 Oct 20;19(20):13577. doi: 10.3390/ijerph192013577 (PMC9603139; doi:10.3390/ijerph192013577)
Supplement: Supplementary file 1 [file ijerph-19-13577-s001.zip › ijerph-1962031-supplementary.pdf]

**Supplementary Material**

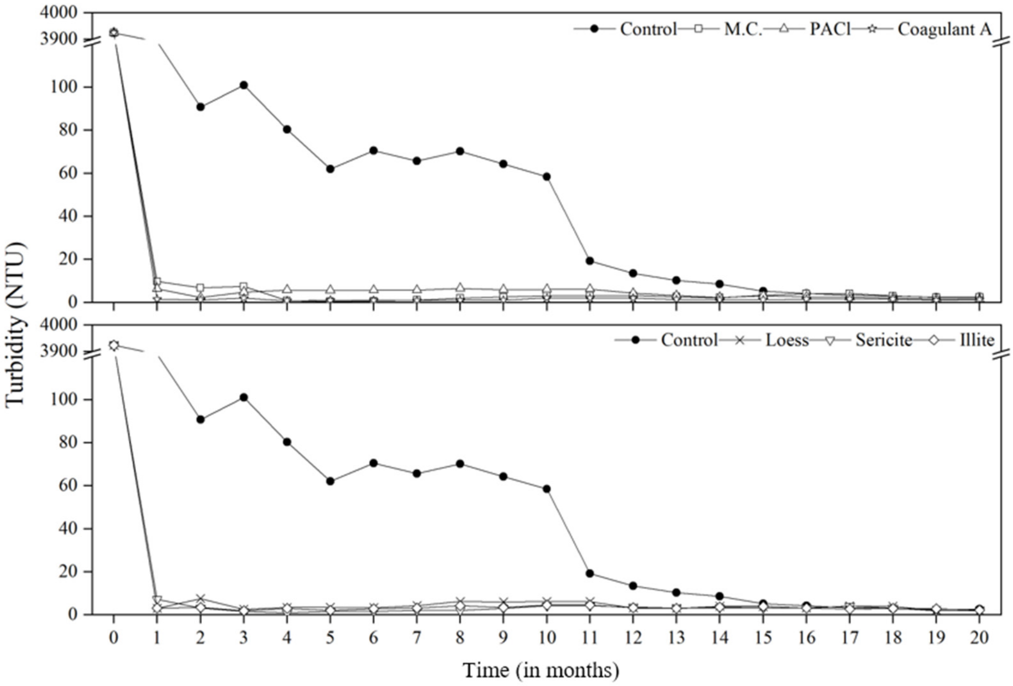


**Figure S1.** Changes in the turbidity values of control and samples treated with coagulants and minerals. MC: mineralized coagulant; PACl: polyaluminum chloride; A: commercial coagulant.

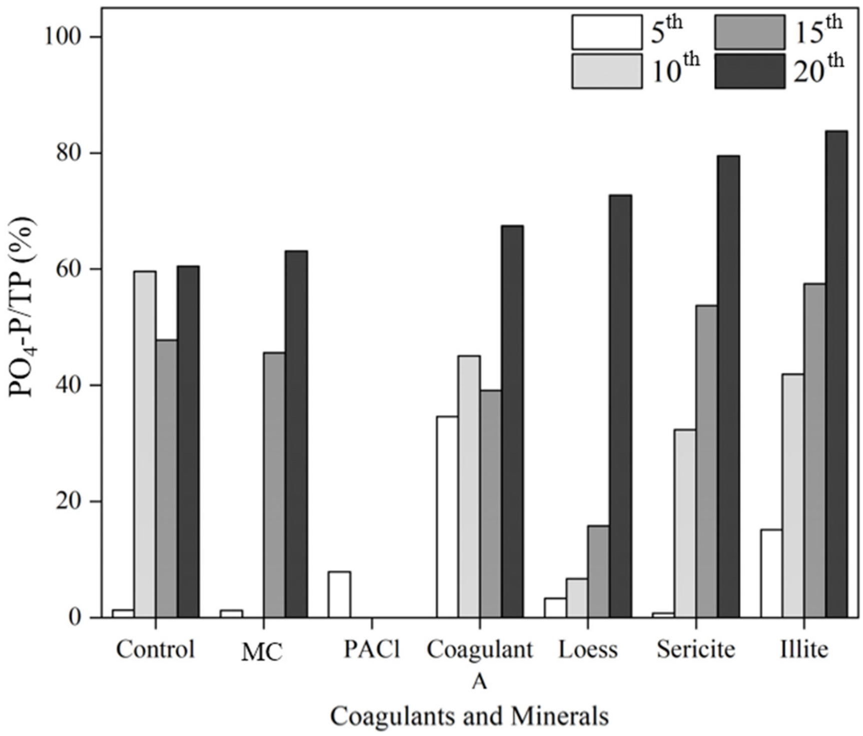


**Figure S2.** Changes in phosphate concentrations in the control and the treatments with various coagulants and minerals. MC: mineralized coagulant; PACl: polyaluminum chloride; A: commercial coagulant.

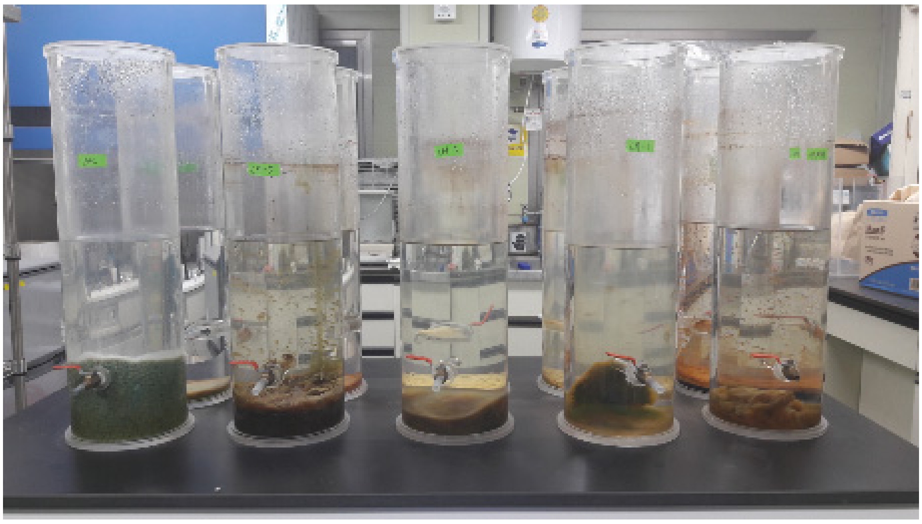

**Figure S3.** The experimental columns used in the study. The photo was taken at the end of the experiment.
